# Types and Distribution of Congenital Heart Defects in Pediatric Patients with Down’s Syndrome: A Retrospective Study

**DOI:** 10.7759/cureus.11133

**Published:** 2020-10-24

**Authors:** Asmaa Ghmaird, Tahani Nasser Alrashidi, Yazeed S Alqahtani, Ahmed H Alanazi, Yousef A Alaenzi, Ahad M Almohammadi, Hussam A Alzahrani, Nawaf F Alomrani, Wafaa Altabbish

**Affiliations:** 1 Paediatrics, College of Medicine, University of Tabuk, Tabuk, SAU; 2 General Practice, College of Medicine, University of Tabuk, Tabuk, SAU; 3 Paediatric Cardiology, King Salman Armed Forces Hospital, Tabuk, SAU

**Keywords:** down’s syndrome, congenital heart defect, atrioventricular septal defect, ventricular septal defect

## Abstract

Background

Congenital heart disease (CHD) is common in patients with Down’s syndrome (DS), rendering them at risk of significant mortality and morbidity. However, all patients with confirmed DS must undergo further investigation for a cardiac anomaly early in their lives.

Objective

To define frequency and patterns of CHD among children with DS in Northwest, Saudi Arabia (SA).

Methods

A five-year chart review study was conducted in Northwest SA between January 2015 and June 2019. We included all children referred to the pediatric cardiology clinic with a confirmed diagnosis of DS and CHD. Children were identified in the clinic’s database, and charts were reviewed retrospectively.

Results

Among 851 patients with CHD, 53 were identified with DS. Male patients represented 62.3%, while female patients represented 37.7%. The median patients’ age at the time of diagnosis was two days, with most patients diagnosed before 28 days old (67.9%). This study found that atrial septal defect (ASD) and patent ductus arteriosus (PDA) were the most common isolated lesions (37% of isolated cases), while the most common combined lesions were ASD, ventricular septal defect (VSD), and PDA, as well as combined ASD and VSD. Female gender was significantly associated with higher percentage of VSD (p = 0.031) more than male. While male patients had significantly higher percentages of ASD and valvular anomalies than female patients (p = 0.019 and 0.033, respectively).

Conclusions

The patient’s gender was significantly associated with some types of CHD. Both ASD and valvular lesions were more common among males, while VSD was more common among female patients, no gender differences were detected regarding the other types of CHD.

## Introduction

Down’s syndrome (DS) is considered as the most frequent chromosomal anomaly to affect newborns, with a global incidence ranging from 1/600 to 1/1000. In Saudi Arabia (SA), the incidence of DS is 1/554 for live births [1-3]. This syndrome results from an additional chromosome 21, thus known as trisomy 21 [3,4]. Clinically, a DS diagnosis depends on dysmorphic features and karyotyping [1, 3].

DS has been associated with many congenital defects, congenital heart disease (CHD) being one of them [1]. Annually, 1.35 million people are born with CHD, with 4-10% associated with DS [4]. The atrioventricular septal defect (AVSD) appears to be the most frequent type of CHD in children with DS [1]. CHD is considered to be the most common cause of mortality in DS patients below the age of two years [5]. Early detection of CHD in patients with DS might prevent various complications. In SA, especially in Tabuk, there was no recent study on the distribution and frequency of CHD in children with DS according to gender. This study should contribute to improving the tools for diagnosis as well as increased awareness for healthcare providers. This highlights our aim to define the frequency and patterns of CHD among children with DS in Northwest SA.

## Materials and methods

This was a retrospective chart review study conducted at King Salman Armed Forces Hospital (KSAFH), Tabuk, SA, over a period of five years from January 2015 to June 2019. The KSAFH is a secondary hospital located in northwest SA, with 900 beds, and it considered as the pediatric cardiology referral center in this region. 

The study included all patients younger than 18 years with CHD and diagnosed with DS based on the typical clinical manifestations and confirmed by cytogenetic studies. Patients were excluded in the following conditions: a) older than 18 years; b) diagnosis of DS was not confirmed by cytogenetic studies; c) incomplete data; and d) patients with a questionable diagnosis of CHD. Ethical approval was obtained from the Research Ethics Committee at KSAFH, SA. To ensure patient confidentiality and anonymity, each patient was assigned a numeric code on his/her respective datasheet. Furthermore, the information was viewed and accessed by only the research members. Children were identified from the clinic’s database, and their charts were reviewed through their MRNs. A total of 851 children were referred to the pediatric cardiology clinic where a diagnosis of CHD was confirmed; however, only 53 patients were diagnosed with CHD and DS. A datasheet was used to collect and organize study variables, including the total number of cases, cases with CHD associated with DS, patient’s age at referral, sex, echocardiography findings [ASD, VSD, PDA, AVSD, Tetralogy of Fallot (TOF), cardiomegaly, valvular abnormalities], isolated or combined heart disease, and types of management. Data were saved in an appropriately designed Excel spreadsheet.

Statistical analysis

Data were analyzed using the Statistical Package for Social Science (IBM SPSS, Armonk, NY, USA) version 20. Categorical data were presented as frequency and percentage. Distribution of age (numerical variable) was assessed using Shapiro-Wilk test and was non-normally distributed; hence, median and range were used as summary statistics. Proportion between two groups was analyzed by Chi-square test or the Fisher’s exact test (when the expected count in any cell was found to be less than 5). The p-value was considered significant at p < 0.05. 

## Results

A total of 851 patients with CHD attended the cardiology clinic, with only 53 (6.2%) patients diagnosed with DS. Male patients represented 62.3% (33 patients), while female patients represented 37.7% (20 cases). All studied cases were younger than 18 years, suffering from CHD and diagnosed with DS based on the typical clinical manifestations, confirmed by cytogenetic studies. The median age of patients at the time of diagnosis was two days, ranging from one to 1,800 days. Most patients were diagnosed before 28 days of life (67.9%), followed by patients diagnosed between two to five years old (18.9%), while patients diagnosed between one and two years old represented 3.8%. Regarding the disease condition of the studied cases, only eight had isolated CHD (15.1%), while 45 patients (84.9%) had combined types of CHD. Only 15 cases (28.3%) were managed surgically, while 38 cases (71.7%) were managed by conservative treatment.

Regarding the prevalence of CHD types, the most common was ASD (81.1% of studied cases), followed by PDA (67.9%), valvular abnormality (58.5%), and the least common type was cardiomegaly (7.5%). Regarding valvular abnormality of the studied cases, 37.7% of patients suffered from tricuspid regurgitation, 9.4% suffered from mitral regurgitation, while each of pulmonary stenosis and aortic regurgitation were found in 7.5% of cases. The least common type was aortic stenosis (1.9%).

The present study found that ASD and PDA represented the most common isolated lesions (37% of isolated cases), while the most common combined lesions were ASD, VSD, and PDA, followed by combined ASD & VSD (Table 1).

**Table 1:** Age at diagnosis, gender, type of congenital heart disease, and case management (total n=53). ASD, atrial septal defect; VSD, ventricular septal defect; PDA, Patent ductus arteriosus; AVSD, Atrioventricular septal defect; TOF, Tetralogy of Fallot.

Parameter		n		%
Age at diagnosis	Less than 28 days	36	67.9%	
	29 days to 1 year	5	9.4%	
	More than 1 to 2 years	2	3.8%	
	2-5 years	10	18.9%	
	Median	2 days		
	Range	1-1,800 days		
Gender	Male	33		62.3%
	Female	20		37.7%
Types of management	Conservative	38		71.7%
	Surgical	15		28.3%
Types of CHD	ASD	43		81.1%
	VSD	27		50.9%
	PDA	36		67.9%
	AVSD	12		22.6%
	Valvular abnormalities	31		58.5%
	Aortic Regurgitation	4		7.5%
	Aortic Stenosis	1		1.9%
	Mitral Regurgitation	5		9.4%
	Pulmonary Stenosis	4		7.5%
	Tricuspid Regurgitation	20		37.7%
	TOF	5		9.4%
	Cardiomegaly	4		7.5%

Regarding the relation between CHD and the gender of DS patients, the present study found that there was a significant association between patient gender and some types of CHD. Male patients had significantly higher percentages of ASD (90.9%) and valvular abnormalities (69.7%) than female patients (65% and 40%, p = 0.019 and 0.033, respectively). On the other hand, female patients had significantly higher percentage of VSD (70%) and non-significantly higher percentage of PDA (80%) than males (39.4% and 60.6%, p = 0.031 and 0.143) (Table 2).

**Table 2:** Association of gender with the type of congenital heart disease in the studied patients (total n=53). ASD, atrial septal defect; VSD, ventricular septal defect; PDA, Patent ductus arteriosus; AVSD, Atrioventricular septal defect; TOF, Tetralogy of Fallot.

	Gender				Chi-square test	
	Female (n = 20)		Male (n = 33)			
	n	%	n	%	X^2^	P-value
ASD	13	65.0%	30	90.9%	5.461	0.019*
VSD	14	70.0%	13	39.4%	4.668	0.031*
Valvular Abnormalities	8	40.0%	23	69.7%	4.523	0.033*
PDA	16	80.0%	20	60.6%	2.150	0.143
AVSD	3	15.0%	9	27.3%	1.071	0.301
TOF	1	5.0%	4	12.1%	0.739	0.390
Cardiomegaly	2	10.0%	2	6.1%	0.277	0.599

## Discussion

CHD is one of the birth defects that might be seen in patients with DS and considered the most common cause of mortality in DS patients below the age of two years [1,5-6]. Early detection of CHD in patients with DS might reduce morbidity and mortality. The present study was conducted in Tabuk to assess the frequency and gender distribution of CHD in children with DS. We found that 6.2% of the referred CHD cases had a confirmed diagnosis of DS. In several studies, CHS has been reported in 4-10% and is often associated with DS [5].

In the current study, male patients represented 62.3%, while female patients represented 37.7%. The percentage of male and female patients were different from those reported in the study by Al-Jarallah reported that 56.3% were males and 43.6% were females and Almawazini et al. reported that 43.2% of cases were males and 56.8% were females [1,7].

The most common CHD was ASD (81.1% of studied cases), followed by PDA (67.9 %), valvular abnormality (58.5%), and the least common type was cardiomegaly (7.5%). Both ASD and PDA represented the most common isolated lesions (37% of isolated cases), while the most common combined lesions were combined ASD, VSD, and PDA, followed by combined ASD and VSD. These results coincided with those reported by Abduljawad and his colleagues in Jeddah, SA, where they found ASD and PDA to be the most common CHD in DS patients [8]. On the other hand, PDA was the most prevalent type in Jeddah, as seen by Al-Aama [9]. While AVSD was the most prevalent in Albaha and Al-Madinah [1,3]. On the other hand, a study in Riyadh reported that the most common CHD was VSD [7] (Table 3).

**Table 3:** Prevalence of CHD with DS in different SA regions. PDA, Patent ductus arteriosus; ASD, atrial septal defect; VSD, ventricular septal defect; AVSD, atrioventricular septal defect; TOF, Tetralogy of Fallot; PS, pulmonary stenosis; TR, tricuspid regurgitation; MR, mitral regurgitation; AR, aortic regurgitation; AS, aortic stenosis.

CHD	Current study		Al-Jarallah [7]		Al-Aama et al. [9]		Almawazini et al. [1]		Morsy et al. [3]		Abduljawad et al. [8]	
	Tabuk		Riyadh		Jeddah		Albaha		Al-Madinah		Jeddah	
	N	%	N	%	N	%	N	%	N	%	N	%
PDA	36	67.9	4	7.4	44	47.8	23	18.4	15	8.5	46	41.8
ASD	43	81.1	14	26.0	38	41.3	14	11.2	38	21.4	57	51.8
VSD	27	50.9	23	42.6	27	29.3	16	12.8	19	10.7	25	22.7
AVSD	12	22.6	8	14.8	11	12.0	61	48.8	72	40.7	19	17.3
TOF	5	9.4	2	3.7	2	1.5	2	1.6	4	2.3	4	3.6
Cardiomegaly	4	7.5					2	1.6				
PS	4	7.5	1	1.9	2	1.5	1	< 1				
TR	20	37.7			31	33.7	4	3.2				
MR	5	9.4			10	10.8	2	1.6				
AR	4	7.5					1	< 1%				
AS	1	1.9										

In agreement with the findings of our study, de Rubens Figueroa et al. in Mexico City and Elmagrpy et al. in Libya found the most common CHD to be ASD in their series [4,10]. In contrast, Ali and Benhaourech et al. in Sudan as well as Nisli et al. in Turkey reported that AVSD was the most common [11-13]. El-Gilany et al. in Egypt, Salih et al. in Iraq, Somasundaram and Ramkumar in India, and Freeman et al. in USA found that VSD was the most common CHD [2,14-16] (Table 4). 

**Table 4:** Prevalence of CHD in different countries. PDA, Patent ductus arteriosus; ASD, atrial septal defect; VSD, ventricular septal defect; AVSD, atrioventricular septal defect; TOF, Tetralogy of Fallot; PS, pulmonary stenosis; TR, tricuspid regurgitation; MR, mitral regurgitation; AR, aortic regurgitation; AS, aortic stenosis.

CHD	Current study		de Rubens Figueroa et al. [10]		Benhaourech et al. [12]		Somasundaram & Rumkumar [15]		El-Gilany et al. [2]		Ali [11]		Nisli et al. [13]		Salih et al. [14]		Freeman et al. [16]		Elmagrpy et al. [4]	
	Tabuk		Mexico City		Morocco		India		Egypt		Sudan		Turkey		Iraq		USA		Libya	
	N	%	N	%	N	%	N	%	N	%	N	%	N	%	N	%	N	%	N	%
PDA	36	67.9	33	21	31	16.7	8	14.5	28	4.4	6	7	15	3.6	32	18.4			28	5
ASD	43	81.1	39	24	37	19.9	11	20	80	12.6	2	2.5	69	16.7	14	8	273	18.6	125	23
VSD	27	50.9	35	22	40	21.5	19	34.5	253	39.8	19	23	68	16.5	68	39.3	282	19.2	76	14
AVSD	12	22.6	9	6	54	29			104	16.4	38	48	141	34.2	37	21.3	252	17.2	103	19
TOF	5	9.4			10	5.4			13	2	5	6	2	0.48	6	3.4	39	2.7	13	2
PS	4	7.5			2	< 1			2	0.3			12	2.9						
TR	20	37.7																		
MR	5	9.4																		
AR	4	7.5																		
AS	1	1.9											8	1.9	1	0.5				

The pattern of CHD varied widely among the studies which may be attributed to differences in study design, as well as differences related to population that were reported as potential risk factors for CHD, including ethnicity, age of mother at pregnancy, maternal smoking [17-18]. In addition to these were maternal deficiencies of folic acid and vitamins, and available prenatal care [2,19]. Another potential explanation is differences in the ratios of male and female patients included in the studies as a significant association between gender and both the prevalence and types of CHD has been described [20]. Moreover, there appears to be a downward trend in the prevalence of CHD in DS patients as reported by Bergström et al. in Sweden and El-Gilany et al. in Egypt, which they explained by improved prenatal care in both countries and selective abortion of fetuses with DS in Sweden [2,18]. 

Previous studies reported controversial results with regards to the association of gender with the type of CHD. The prevalence of CHDs among female patients with DS was reported to be higher than males with DS [18,21-22]. In contrast, Somasundaram and Ramkumar reported that males suffered from CHD more than females [15-16].

In the present study, female gender was significantly associated with higher percentage of VSD (70%) and non-significantly associated with higher percentage of PDA (80%) than males (39.4% and 60.6%, p = 0.031 and 0.143). We found also that male patients had significantly higher percentages of ASD (90.9%) and valvular anomalies (69.7%) than female patients (65% and 40%, p = 0.019 and 0.033, respectively. With regards to the association of gender with some types of CHD, the results of previous studies also showed wide variations. Freeman et al. found that VSD was more common in males whereas ASD was more prevalent in females [16]. Morris et al. found a higher frequency of VSD, TOF, and ASD in male than female patients with DS, while AVSD was predominantly encountered in females [21]. Santoro et al. reported that AVSD, ASD, and VSD were significantly more frequent in females in their series of DS patients [22]. In a meta-analysis by Diogenes et al. which included 12 articles, AVSD was significantly highly prevalent in females compared to males, with no significant differences in prevalence of other CHD types between the two genders [20].

The explanation of this observed gender difference is difficult to clarify. However, one potentially plausible explanation could be that the death of male fetuses with DS may be higher than female fetuses, hence the higher prevalence of CHD in female live born cases. The proof of this hypothesis requires full autopsy of dead fetuses with DS, which is not routinely performed [20, 23].

Regarding the studied cases, only eight had isolated CHD (15.1%), while 45 patients (84.9%) had combined CHD; this was in contrast with Salih et al. who found that isolated cardiac defects represented 73.3% of the total cases [14]. 

The present study bears some points of strength, being the first of its type in Tabuk region, SA and was conducted in a referral center over an adequate duration (5 years). However, our study was limited by its retrospective nature so data on the potential risk factors that may have resulted in the depicted pattern of CHD were not available for analysis. In addition, the sample size was small as it was a single-center study. Future studies should be designed on a larger scale including larger sample size. Prospective results are recommended, preferably multi-center, to investigate the potential risk factors which may determine the type of CHD in DS patients.

## Conclusions

In the present study, 6.2% of CHD was associated with DS. The present study concluded that the most prevalent types of CHD in DS patients was ASD, followed by PDA, with the most common combined lesions being ASD, VSD, and PDA, followed by combined ASD & VSD. Both ASD and valvular lesions were more common among males, while VSD was more common among female patients. No gender differences were detected regarding the other types of CHD.

## References

[1] Almawazini AM, Sharkawy AA, Eldadah OM, Sumaily YA, Alamary TY (2017). Congenital heart diseases in down syndrome children at Albala area, Saudi Arabia. Neonat Pediatr Med.

[2] El- Gilany AH, Yahia S, Wahba Y (2017). Prevalence of congenital heart diseases in children with Down syndrome in Mansoura, Egypt: a retrospective descriptive study. Ann Saudi Med.

[3] Morsy MM, Algrigri OO, Salem SS (2016). The spectrum of congenital heart diseases in down syndrome. A retrospective study from Northwest Saudi Arabia. Saudi Med J.

[4] Elmagrpy Z, Rayani A, Shah A (2011). Down syndrome and congenital heart disease: why the regional difference as observed in the Libyan experience?. Cardiovasc J Afr.

[5] Santos F, Croti UA, Marchi CH (2019). Surgical treatment for congenital heart defects in down syndrome patients. Braz J Cardiovasc Surg.

[6] Boussouf K, Zaidi Z, Amrane M (2017). Study of congenital heart diseases in patients with Down syndrome in Algeria. East Mediterr Health J.

[7] Al-Jarallah AS (2009). Down's syndrome and the pattern of congenital heart disease in a community with high parental consanguinity. Medical Science Monitor.

[8] Abduljawad EM, AlHarthi A, AlMatrafi SA, Hussain M, Shawli A, Waggass R (2020). The prevalence of congenital heart diseases in syndromic children at King Khalid National Guard Hospital from 2005 to 2016. Cureus.

[9] Al-Aama JY, Bondagji NS, El-Harouni AA (2012). Congenital heart defects in Down syndrome patients from western Saudi Arabia. Saudi Med J.

[10] de Rubens Figueroa J, del Pozzo Magaña B, Pablos Hach JL (2003). Heart malformations in children with Down syndrome. Rev Esp Cardiol (English Edition).

[11] Ali SK (2009). Cardiac abnormalities of Sudanese patients with Down’s syndrome and their short-term outcome. Cardiovasc J Afr.

[12] Benhaourech S, Drighil A (2016). Congenital heart disease and Down syndrome: various aspects of a confirmed association. Cardiovasc J Afr.

[13] Nisli K, Oner N, Candan S (2008). Congenital heart disease in children with Down’s syndrome: Turkish experience of 13 years. Acta cardiologica.

[14] Salih AF (2011). Congenital heart disease in down syndrome: experience of Kurdistan of Iraq. Duhok Medical Journal.

[15] Somasundaram A, Rumkumar P (2018). Study on congenital cardiac defects of Down syndrome children. J Pediat Infants.

[16] Freeman SB, Bean LH, Allen EG (2008). Ethnicity, sex, and the incidence of congenital heart defects: a report from the National Down Syndrome Project. Genet Med.

[17] Alverson CJ, Strickland MJ, Gilboa SM, Correa A (2011). Maternal smoking and congenital heart defects in the Baltimore-Washington Infant Study. Pediatrics.

[18] Bergström S, Carr H, Petersson G (2016). Trends in congenital heart defects in infants with Down syndrome. Pediatrics.

[19] Bean LJ, Allen EG, Tinker SW (2011). Lack of maternal folic acid supplementation is associated with heart defects in Down syndrome: a report from the National Down Syndrome Project. Birth Defects Res A Clin Mol Teratol.

[20] Diogenes TCP, Mourato FA, de Lima Filho JL, Mattos SdS (2017). Gender differences in the prevalence of congenital heart disease in Down’s syndrome: a brief meta-analysis. BMC Medical Genetics.

[21] Morris JK, Garne E, Wellesley D (2014). Major congenital anomalies in babies born with Down syndrome: a EUROCAT population-based registry study. Am J Med Genet A.

[22] Santoro M, Coi A, Spadoni I (2018). Sex differences for major congenital heart defects in Down Syndrome: a population based study. Eur J Med Genet.

[23] Dolk H, Loane M, Garne E, European Surveillance of Congenital Anomalies (EUROCAT) Working Group (2011). Congenital heart defects in Europe: prevalence and perinatal mortality, 2000 to 2005. Circulation.

